# Auditory reafferences: the influence of real-time feedback on movement control

**DOI:** 10.3389/fpsyg.2015.00069

**Published:** 2015-01-30

**Authors:** Christian Kennel, Lukas Streese, Alexandra Pizzera, Christoph Justen, Tanja Hohmann, Markus Raab

**Affiliations:** ^1^Institute of Psychology, German Sport University Cologne, Cologne, Germany; ^2^Institute of Sports Science, University of Stuttgart, Stuttgart, Germany; ^3^Department of Applied Sciences, London South Bank University, London, UK

**Keywords:** reafference, action perception, feedback, track and field, movement sound, motor control, motor learning

## Abstract

Auditory reafferences are real-time auditory products created by a person’s own movements. Whereas the interdependency of action and perception is generally well studied, the auditory feedback channel and the influence of perceptual processes during movement execution remain largely unconsidered. We argue that movements have a rhythmic character that is closely connected to sound, making it possible to manipulate auditory reafferences online to understand their role in motor control. We examined if step sounds, occurring as a by-product of running, have an influence on the performance of a complex movement task. Twenty participants completed a hurdling task in three auditory feedback conditions: a control condition with normal auditory feedback, a white noise condition in which sound was masked, and a delayed auditory feedback condition. Overall time and kinematic data were collected. Results show that delayed auditory feedback led to a significantly slower overall time and changed kinematic parameters. Our findings complement previous investigations in a natural movement situation with non-artificial auditory cues. Our results support the existing theoretical understanding of action–perception coupling and hold potential for applied work, where naturally occurring movement sounds can be implemented in the motor learning processes.

## INTRODUCTION

Sounds can help athletes perform better (Agostini et al., 2004). Especially in fast and short or rhythmic movements, auditory feedback might be superior to visual feedback (MacPherson et al., 2009). Many, if not all, sports skills can be considered rhythmic in nature (Gallahue and Donnelly, 2003), and rhythm (or temporal invariance of movement components) is a crucial aspect of many sports skills. Coaches also report using auditory information for error detection as well as a kind of augmented feedback (Chen, 2001). But there has been little theoretical or empirical research to determine if the auditory information produced by the movement itself is also used for motor control during movement execution. In the current study, we investigated for the first time the influence of naturally occurring auditory real-time feedback (i.e., perception) on the movement execution (i.e., action) of a complex movement task. Therefore we used a hurdling task, which is characterized by a typical auditory rhythm. Furthermore, the hurdling task represents on the one hand a complex movement, which allows conclusions for the applied field, but on the other hand a movement with a highly standardized motion sequence, given the predetermined number of steps and distance between two hurdles.

One description of this bidirectional link of action and perception is the common coding theory (Prinz, 1997; Hommel et al., 2001; Schütz-Bosbach and Prinz, 2007). The core assumption is that action and perception share common mechanisms and thus are functionally equivalent. However, the actual phenotypes of these mechanisms remain uncertain. In a framework proposed by Schubotz (2007) these common mechanisms are seen as representations in the form of internal models, and the dynamic aspect of action–perception coupling becomes apparent. Internal models simulate the consequence of an action (Miall and Wolpert, 1996; Wolpert and Flanagan, 2001) and stress the role of sensory feedback in motor control (Desmurget and Grafton, 2000). In more detail, internal models help explain how movement is controlled by comparing expected feedback and actual feedback. It has been proposed that for every motor command, there is a copy with predicted feedback (Blakemore et al., 2000). The de facto feedback, called reafference (Von Holst and Mittelstaedt, 1950), is compared to this predicted feedback. This comparison and any adjustment is the basis of movement calibration. The reafference delivers interoceptive (mostly sensorimotor) and exteroceptive (mostly visual and auditory; Schubotz, 2007) information.

So far, the role of sensorimotor and visual feedback has been investigated much more than the influence of naturally occurring auditory feedback, yet the natural auditory feedback that automatically accompanies movements is highly informative (Kennel et al., 2014a). In addition, dynamic temporal movements can be depicted through sound precisely (MacPherson et al., 2009). Furthermore, auditory feedback has no perspective problem (compare this to visual feedback; many existing studies in the visual domain used stimulus material from a third-person perspective, which does not match subjects’ self-perception). Nonetheless, it remains unclear how real-time auditory feedback affects motor control. For effective motor control, the sensory feedback should determine not only the position of the involved limbs (which is mostly done by other senses, e.g., kinesthetic, visual) but also movement characteristics such as frequency and amplitude, which are provided by auditory movement information.

Studies have investigated action–perception coupling by conducting “offline” perceptual experiments. Offline refers to temporally separated perception, not concurrent with action execution, of previously recorded movements. When the influence of action on perception has been examined, results have shown that producing an action primes the perceptual sensitivity, meaning that higher motor experience leads to a more accurate perceptual performance. This research was mainly done as visual perception experiments (for a review, see Schütz-Bosbach and Prinz, 2007). However, studies from the auditory domain show comparable results. For instance, even simple clapping sounds (Flach et al., 2004) enabled stable self-recognition. This ability remained even when the salience of the stimuli was reduced. Also classical piano music is perceived more accurately with matching motor experience. Artificial modifications in previously recorded musical excerpts from different authors were detected better when the excerpts were self-produced (Repp and Keller, 2010). Current research shows similar findings with the help of human movement sounds. For instance, participants were able to identify self-generated movement sounds 64% of the time, whereas strangers’ sounds were identified with an accuracy of 47% (Murgia et al., 2012; Kennel et al., 2014a). In contrast, it is not yet known whether natural sounds, produced as a by-product of movement, affect action execution. Because auditory reafferences are closely aligned with representations, which are needed to control movement, this study has the potential to advance both the development of theory and practical applications.

Our main aim in the present study was to examine the influence of constant auditory feedback on the performance of a complex movement task, hurdling, which is characterized by a typical rhythmic structure and a predetermined number of steps. By using a complex movement task that exists in the same form in competitions, we hoped the findings would be useful in applied settings. The results of previous studies (Aschersleben et al., 2001; Menzer et al., 2010) and theoretical approaches (Miall and Wolpert, 1996; Schubotz, 2007) led us to hypothesize that missing auditory feedback (1a) would decrease performance (overall time) and (1b) increase the variance in motion sequences (kinematics) in a complex movement task in comparison to a control condition. Furthermore, we hypothesized that original but delayed auditory feedback would also have a negative influence on (2a) performance (overall time) and (2b) variance in motion sequences (kinematics), caused by the feedback frequently overlapping in time with the successive units of motor performance (Chase et al., 1961).

## MATERIALS AND METHODS

### PARTICIPANTS

Twelve female and eight male students aged 21.9 ± 2.6 years participated in the study in return for financial compensation of €10 per hour. The mean body height and weight were 175.0 ± 6.2 cm and 68.4 ± 9.3 kg. Participants were track and field athletes, recruited from the athletics education program at the local sport university. Within this athletics education program, all participants passed a practical test that complied with the requirements of the experiment. The participants did not know each other, had no further information on the content of the study, and self-reported having normal hearing. The investigation was approved by the local ethics committee and performed according to the Helsinki Declaration.

### TASK

The experiment was run on an indoor Tartan track at the local university. The movement task was the clearance of four hurdles with a predetermined number of steps: four between each hurdle and eight steps from the start (out of a starting block) to the first hurdle. The distance to the first hurdle was 13.00 m and there were 8.50 m between each of the following hurdles (1–2, 2–3, 3–4). These dimensions (official International Association of Athletics Federations 100-m women’s hurdles) turned out to be optimal in a pilot study. The height of the hurdles (ERHARD SPORT, Geslau, Germany) was 91.4 cm. There was no acoustical start signal, so the participants could start whenever they were ready. We recorded four valid attempts (correct number of steps and clearance of the hurdles without contact with the hurdle) of every participant for each of the three conditions.

### APPARATUS

Time and kinematic data were collected to estimate the performance of the movement task. The overall time was measured by double light barriers in order to reduce measurement errors. The starting point (first light barrier) was placed 5 m after the start from a starting block, to disregard individual reaction time. The end (second light barrier) was placed at 40 m, directly after the last hurdle. The two-dimensional kinematic data were recorded on the third hurdle (peak velocity) using a high-speed camera (Casio EX-FH100) with a 120 frames/s recording speed, a resolution of 640 × 480 pixels, and calibration with a 2 × 2-m square. The considered parameters, essential for hurdling performance, were flight time, flight distance, flight height, horizontal flight velocity, step duration (the time that the foot was in contact with the floor either before or after the hurdle), and angle of the hurdle step (lead leg and trailing leg). Performance on these parameters turned out to conform to the average skills in a pilot study.

### FEEDBACK APPARATUS

**FIGURE 1:**
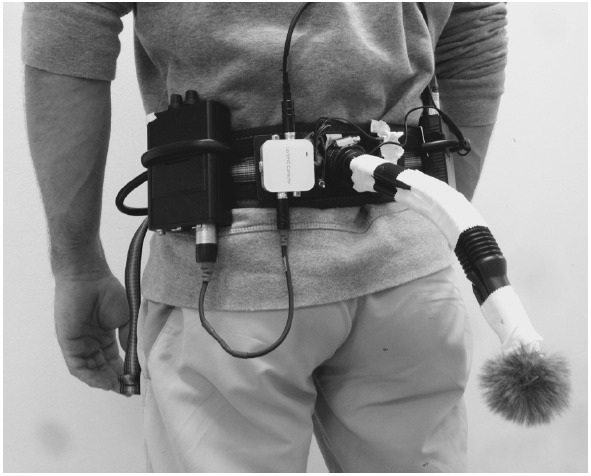
**The technical device to record sounds online and provide them with adjustable time-delay**.

The purpose of the online feedback system was to record the movement sounds and present them to the participants online through headphones, in a natural or manipulated way. The system consisted of a microphone, a battery supply unit, an audio delay converter, a headphone amplifier, and headphones (see Figure 1 for the array). The condenser microphone (C 417 L, AKG, Vienna, Austria) with omnidirectional polar pattern [sensitivity at 1 kHz: 10 mV/Pa (–40 dBV re 1V/Pa); maximum sound pressure level (SPL) for 1%/3% THD: 118/126 dB SPL] was placed on the lower back to record the step sounds. It was protected with an acrylic cover to reduce background noises from the wind. Power was supplied by a mobile battery supply unit (B 29 L, AKG, Vienna, Austria). For the condition with delayed auditory feedback, an audio delay converter (DCT-18, SpeaKa Professional, Hirschau, Germany) was switched on. The headphone amplifier (Hardwired In-Ear Body Pack, Fischer Amps, Osterburken, Germany) connected the microphone delay system with the output in the microphones. Closed diffuse-field studio headphones (DT 770 M, beyerdynamic, Heilbronn, Germany) were used to present the movement sounds. They are characterized by their high noise attenuation (35 dBA), a high SPL (105 ndB), and a total harmonic distortion of less than 0.2%. The technical components were connected by high qualitative XLR or Cinch connectors. The complete array was fixed to an individually adjustable climbing harness worn around the hips, which provided a very good fit for each participant. The total weight of the technical equipment was 1.4 kg.

### DESIGN AND PROCEDURE

The experiment had a within-subject design consisting of three conditions (control, white noise, and delayed) and four trials per condition in a randomized order. Each participant was invited individually. After the movement task was explained, the mobile online feedback system was wired onto the body of the participant. To become accustomed to the technical equipment, every participant had three warm-up trials.

The three conditions differed with regard to the auditory feedback. In the control condition, the conventional sound produced by the movement (step sounds) was given. In the delayed condition, the auditory feedback was presented with a delay of 180 ms. This time was chosen to ensure self-attribution, following Menzer et al. (2010). In the white noise condition, the auditory feedback was masked by white noise. The task in all conditions was to pass the hurdles as fast as possible. The recovery time between each condition was 3 min. The overall duration of the experiment was about 1 h.

### STATISTICAL ANALYSIS

Overall time and kinematic data of the movement task were determined as the mean of the four trials per condition. We performed an analysis of variance (ANOVA) with repeated measures on the three-level factor condition (control, white noise, delayed). We checked for sphericity with Mauchly’s test. If a violation was found, Greenhouse–Geisser correction was used to adjust the level of significance. There were no missing values and no removed outliers. Bonferroni *post hoc* analysis was used to compare the means. Eta-squared effect sizes were additionally calculated to estimate the magnitude of the effects. A significance criterion of *p* = 0.05 was established for all results reported.

## RESULTS

### EFFECTS OF AUDITORY FEEDBACK ON MOVEMENT PERFORMANCE (OVERALL TIME)

**FIGURE 2:**
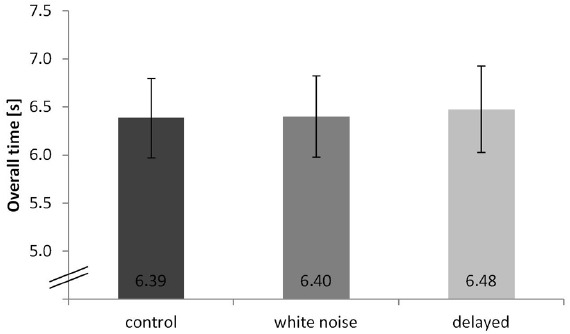
**Overall time of the hurdling task—different conditions displayed in seconds. Error bars indicate the standard deviation**.

We found a significant effect of condition on performance (overall time), depending on the auditory feedback during the movement execution, *F*(2,18) = 7.85, *p* = 0.006, η^2^ = 0.292. A Bonferroni-corrected pair-wise comparison between the delayed and control condition indicated a significant difference (*p* = 0.008). No significant difference was found between the delayed and the white noise condition (*p* = 0.075) or between the white noise and the control condition (*p* = 0.593). Results are highlighted in Figure 2.

**FIGURE 3:**
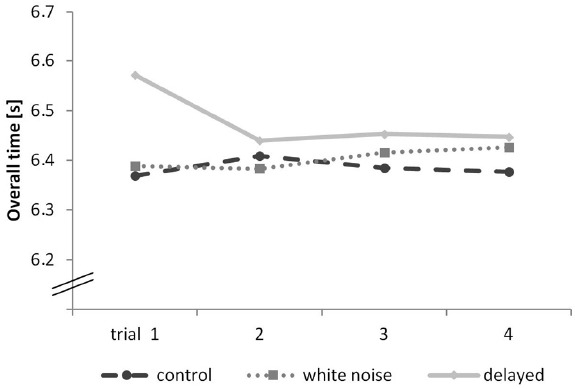
**Overall time progress of each trial of the hurdling task—different conditions displayed in seconds**.

However, a closer inspection of the movement performance (overall time) shows that this difference is mainly caused by the results of the first trial. A repeated-measures ANOVA with condition as the between-subjects factor and trial as the within-subject factor and accepted sphericity revealed a significant main effect of condition on performance (overall time), *F*(2,18) = 10.58, *p* < 0.001, η^2^ = 0.358. A Bonferroni-corrected pair-wise comparison showed significant differences between the delayed and the control condition (*p* = 0.005) as well as between the delayed and the white noise condition (*p* = 0.005). There was no significant difference between the white noise and the control condition (*p* > 0.999). It has to be mentioned that 0.2 s on a track length of 40 m is an enormous difference in hurdling. The results are shown in Figure 3.

### EFFECTS OF AUDITORY FEEDBACK ON MOVEMENT STABILITY (KINEMATICS)

**FIGURE 4:**
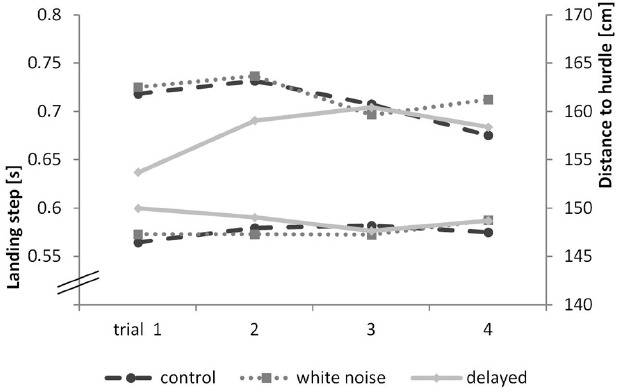
**Kinematic progress of each trial of the hurdling task—landing step is shown in seconds (on the bottom), distance to hurdle in cm (on the top)**.

Our second goal was to examine if delayed or missing auditory feedback influences the variance in motion sequences (movement stability). To rule out possible learning effects and coping strategies, we analyzed the first trial. The parameters we considered were the duration of the landing step (in seconds) and the position (distance to hurdle in centimeters) of the hurdling step (last step before the hurdle). A short landing step close to the hurdle is considered optimal. A repeated-measures ANOVA with accepted sphericity showed a significant effect of condition for the landing step, *F*(2,18) = 3.93, *p* = 0.028, η^2^ = 0.171, and the hurdling step, *F*(2,18) = 5.31, *p* = 0.009, η^2^ = 0.218. A Bonferroni-corrected pair-wise comparison for the landing step revealed a significant difference between the delayed and the control condition (*p* = 0.007), but not between the delayed condition and the white noise condition (*p* = 0.283) or the white noise and the control condition (*p* > 0.999). Pair-wise comparison for the hurdling step showed a significant difference between the delayed and the control condition (*p* = 0.047) but not between the delayed and the white noise condition (*p* = 0.063) or the white noise and the control condition (*p* > 0.999). We found no significant differences in the other kinematic parameters (i.e., flight time, flight distance, flight height, horizontal flight velocity, step duration, and angle of the hurdle step). Results are highlighted in Figure 4.

## DISCUSSION

Our aim in the present study was to investigate the influence of perception on motor performance via an experiment with modulated auditory feedback of naturally occurring movement sounds. Results show that temporally delayed auditory feedback led to a significant decreased performance in a complex movement task. This decrease mainly occurred in the first of four trials. In a situation without auditory feedback (blocked by white noise), this deterioration was not present. Regarding variance in motion sequences, we found significant differences for the parameters landing step and hurdling step. These differences also mainly occurred in the first trial.

Our findings support the theory of action–perception coupling (Schütz-Bosbach and Prinz, 2007) and are in line with findings from the visual (Calvo-Merino et al., 2005; Loula et al., 2005) as well as the auditory (Murgia et al., 2012; Kennel et al., 2014b) and sensorimotor domains (Aschersleben et al., 2001). Our experiment showed for the first time the influence of perceptual processes on action during movement execution (online effects) with the help of modulated but original (normally occurring as a by-product) movement sounds. These findings underpin the understanding that exteroceptive (such as auditory) feedback influences motor control; in particular, auditory reafferences, which are largely similar to internal representations, are used for motor prediction.

As shown by Menzer et al. (2010), humans are sensible to footstep sounds. Especially a person’s own produced step sounds, which have numerous internal representations, seem to deliver information-rich audio–motor cues. This robust interconnection between expected and differently perceived auditory feedback might have caused the movement adaptation and resulting deterioration in the delayed condition (decreased overall time). An influence of delayed auditory feedback has been reported in speech research (Lee, 1950), and also in fine motor experiments. Chase et al. (1961) found that delayed auditory feedback provoked a temporal but also an intensity change in a key-tapping motor task. These changes, however, emerged only when the type of tapping task was complex enough. They argued that temporal complexity is “one of the determinants of the degree to which a task will be disturbed by a delay in sensory feedback” (p. 153). Given its complexity, the informational content of the movement sounds, and its rhythmic structure—our hurdling task appears to be appropriate for gaining knowledge from a vivid environment. However, it cannot be ruled out that it is solely the delay of auditory reafferences and therewith mismatching perception and motor execution, deteriorating the performance. Existing studies already showed, that auditory feedback can have a positive or disturbing influence on motor performance (Baudry et al., 2006; Karageorghis et al., 2009). The post-experimental questionnaire is contradicting this disturbing influence. We asked the participants if they were disturbed by the auditory stimulus through the headphones while performing the movement task on a five point likert scale from 1 = not at all to 5 = very much. The delayed condition revealed a less disturbing influence (1.61) than the white noise condition (2.06) and only a slightly higher influence than the control condition (1.44). In addition, the question arises if sensory (in our study auditory) feedback can be used to control the movement in such a fast and intensive task. In response to this, Desmurget and Grafton (2000) suggested that internal feedback loops should be reconsidered. Fast movement is, according to their view, controlled by continuously updating forward models that integrate sensory input and motor output to evaluate motor commands sent to the periphery (feedback loops). Delayed auditory feedback apparently leads to major discrepancies in expected and actually perceived sensory input, which in turn leads to a changed movement—in our experiment, significantly changed overall time and kinematic parameters. It seems from this that a frequent overlapping in time with successive units of motor performance has the most negative influence on motor performance. The motor system, however, is customizable enough to compensate for and react to the available feedback in such a complex movement situation. From the second trial in the delayed feedback condition and the first trial in the white noise condition (where participants obviously noticed the missing auditory feedback already before the first trial), they might have ignored the useless feedback channel, presumably by focusing on other sensory sources. This phenomenon of interdependency between feedback channels is so far mostly known from long-term effects of deaf participants (Grossenbacher and Lovelace, 2001) and an extreme form known as synesthesia (Finney et al., 2003). However, deeper understanding should be provided by future investigations within the specific domain of complex auditory movement feedback.

The current study contained some limitations. The movement sounds were recorded from a lower back position. A recording from an in-ear position would result in stimulus material closer to internal representations, but because of the simultaneous presentation of the recorded sounds through headphones and the qualitatively better (especially sound volume) recordings with the help of this recording position, we decided on the current method. A post-experiment questionnaire revealed neither alienation effects nor a stranger attribution of the presented stimuli. In all, 65% of the participants recognized a difference between the control and the delayed condition; however, it did not influence the results of the movement task. Moreover, an additional auditory condition with disturbing noise (e.g., traffic noise) could underpin the theoretical interpretation by ruling out the influence of simply disturbing noise compared to delayed reafferences.

In conclusion, we found for the first time that delayed auditory reafferences can influence complex movements. On a theoretical level, this provides extensive evidence from a vivid real-life situation that perception and action are interconnected. When considered together with the results from previous research, it can be assumed that action and perception share some mechanisms or even overlap in their structure. The next step of research could be to shape movement or motor learning with the help of optimized natural sounds while moving. This could be achieved by implementing either the subjects’ own optimized rhythm sounds or expert sounds in athletes’ training.

### Conflict of Interest Statement

The authors declare that the research was conducted in the absence of any commercial or financial relationships that could be construed as a potential conflict of interest.
